# Circular RNA Involvement in the Protective Effect of Human Umbilical Cord Mesenchymal Stromal Cell-Derived Extracellular Vesicles Against Hypoxia/Reoxygenation Injury in Cardiac Cells

**DOI:** 10.3389/fcvm.2021.626878

**Published:** 2021-02-23

**Authors:** Changyi Zhang, Hongwu Wang, Jilin Li, Lian Ma

**Affiliations:** ^1^Departments of Cardiology, Second Affiliated Hospital of Shantou University Medical College, Shantou, China; ^2^Departments of Pediatrics, Second Affiliated Hospital of Shantou University Medical College, Shantou, China; ^3^Department of Hematology and Oncology, Shenzhen Children's Hospital, Shenzhen, China

**Keywords:** circular RNA, AC16 human cardiomyocytes, human umbilical cord mesenchymal stromal cell-derived extracellular vesicles, hypoxia/reoxygenation injury, repair

## Abstract

Human umbilical cord mesenchymal stromal cell-derived extracellular vesicles (HuMSC-EVs) can repair damaged tissues. The expression profile of circular RNAs (circRNAs) provides valuable insights into the regulation of the repair process and the exploration of the repair mechanism. AC16 cardiomyocytes were exposed to hypoxia/reoxygenation (H/R) injury and subsequently cultured with or without HuMSC-EVs (Group T and Group C, respectively). High-throughput RNA sequencing was implemented for the two groups. On the basis of the transcriptome data, gene ontology (GO), Kyoto Encyclopedia of Genes and Genomes (KEGG) pathway, and network analyses were carried out to determine the differential gene expression profiles between the two groups. After screening the circRNA database, the results were proved by quantitative real-time polymerase chain reaction. The survival rate of cardiomyocytes exposed to H/R was increased by treatment with HuMSC-EVs. RNA-seq analysis showed that 66 circRNAs were differentially expressed in cardiomyocytes in the co-cultured group. The cellular responses to hypoxia and to decreased oxygen levels were at the top of the GO upregulated list for the two groups, while the vascular endothelial growth factor signaling pathway, long-term potentiation, and the glucagon signaling pathway were at the top of the KEGG pathway upregulated list for the two groups. In the same samples, the 10 most aberrantly upregulated circRNAs were chosen for further verification of their RNA sequences. Seven of the 10 most aberrant circRNAs were significantly upregulated in the co-cultured group and in the HuMSC-EVs. Our results revealed that upregulated circRNAs were abundant during the repair of damaged cardiomyocytes by HuMSC-EVs, which provides a new perspective for the repair of H/R by HuMSC-EVs.

## Introduction

Myocardial ischemia/reperfusion (I/R) injury is one of the most common cardiac abnormalities following cardiac surgery. To date, there is no effective treatment for I/R injury (1). So, it is urgent need for finding novel therapeutic strategies. Mesenchymal stromal cell (MSC)-based therapy is a promising approach following I/R injury (2, 3). Different mechanisms are associated with this therapy, such as anti-apoptosis, anti-inflammation, and proangiogenesis (4). There is evidence that transplanted MSCs exhibit low survival in the host (5). Hence, the positive effects of MSCs are mainly due to their paracrine effects, especially through extracellular vesicles (EVs) (6).

EVs are nanosized membrane vesicles secreted in an evolutionally conserved manner by all types of cells including MSCs (7). EVs are key mediators of intercellular communication and take part in the transmission of biological information between cells, especially the transport of proteins and various nucleic acids, mainly mRNA and non-coding RNAs (ncRNA) (8). Previous studies have shown that MSC-derived EVs can mimic the biological characteristics of MSCs, such as immune modulation and tissue damage repair (9, 10). Our previous study also showed that human umbilical cord mesenchymal stromal cell-derived EVs (HuMSC-EVs) can protect cardiac cells against hypoxia/reoxygenation (H/R) injury *via* activation of the phosphoinositide 3-kinase (PI3K)/Akt pathway (11). However, the mechanism has not been fully elucidated.

Circular RNA (circRNA) is formed by covalently closed transcripts without a 5' hat structure and 3' poly A tail (12). Because of their closed structure, circRNAs are resistant to exonuclease degradation and have higher stability than linear RNAs (13). Many studies have shown that circRNAs possess various biological functions, including sequestering microRNAs, binding to RNA-binding proteins (RBPs) (14, 15), and even encoding proteins (16). Recently, circRNAs have been implicated in cardiovascular diseases, including myocardial infarction and heart failure (17). However, whether circRNAs play a part in HuMSC-EV-mediated cardiac repair remains unclear.

In the present study, we aimed to investigate the function of circRNAs in the protective effect of HuMSC-EV in H/R injured cardiomyocytes. High-throughput RNA sequencing (RNA-seq) was used to find out the differentially expressed circRNA profiles in damaged cardiomyocytes co-cultured with or without HuMSC-EVs.

## Materials and Methods

### Cell Culture and Extraction of HuMSC-EVs

HuMSCs were isolated as described previously (18). The Institutional Review Board of Shantou University Medical College (Shantou, China) consent the experiment protocol. HuMSCs were obtained from five patients who signed written informed consent forms. The HuMSCs were cultured in Dulbecco's Modified Eagle Medium (DMEM)/F12 medium containing 10% exosome-depleted fetal bovine serum (FBS) and incubated at 37°C in an incubator filled with 5% CO_2_. We use the third passage of the HuMSCs for EV extraction. AC16 human cardiomyocytes were purchased from the American Type Culture Collection (ATCC, Rockville, MD, USA), cultured in DMEM added 10% FBS, 100 U/mL streptomycin, and 100 U/mL penicillin, and incubated at 37°C in an incubator filled with 5% CO_2_. ExoQuick-TC (System Biosciences, CA, USA) was used for HuMSC-EVs isolated as described previously (11).

### Internalization of HuMSC-EVs Into AC16 Human Cardiomyocytes

HuMSC-EVs were labeled with PKH26 (Ca#MIDI26, Sigma-Aldrich, China) for 15 min in the dark at room temperature, washed thrice with PBS, centrifuged at 100,000 × g, and incubated at 4°C for 2 h. The purified EVs were added to the culture medium and incubated with cardiomyocytes for 12 h. Cell nuclei were dyed using 4969-diamidino-2-phenylindole (DAPI, Ca#C1005, Beyotime Biotechnology, Shanghai, China) and the internalization of HuMSC-EVs by the cardiomyocytes was analyzed using the Olympus BX51 confocal microscope (Olympus, Tokyo, Japan).

### *In vitro* H/R Model

To induce hypoxia, the initial culture medium was replaced with buffer (pH 6.2, 137 mM NaCl, 12 mM KCl, 0.49 mM MgCl_2_ × 6H_2_O, 0.9 mM CaCl_2_, 4 mM HEPES, 20 mM Na lactate) (19) and the AC16 human cardiomyocytes were cultured in pure N_2_ gas at 37°C for 3, 6, 12, or 24 h. Afterward, cardiomyocytes were reoxygenated in fresh oxygenated culture medium for 1, 3, 6, and 12 h, respectively (H_3_/R_1_, H_6_/R_3_, H_12_/R_6_, H_24_/R_12_), in an incubator with 5% CO_2_. Three experimental groups were designated as follows: normal group, in which AC16 human cardiomyocytes were cultured under normal conditions (Group N); H/R group (Group C); and H/R+HuMSC-EVs group (Group T), the cardiomyocytes were cultured with HuMSC-EVs (8 μg/mL) for 12 hours prior to H/R. Experiments were carried out in triplicate.

### MTT Assay

3-[4,5-dimethylthiazol-2-yl]-2,5 diphenyl tetrazolium bromide (MTT) assay was used for evaluated the viability of the cardiomyocytes. MTT was added to the culture medium, incubated for 4 h at 37°C, and subsequently solubilized with 150 μL DMSO. The optical density (OD) values were measured at 490 nm using a microplate reader. The viability of the cardiomyocytes was calculated as follows: viability (%) = (OD of assay—OD of blank) / (OD of control—OD of blank) × 100%.

### Western Blot

Cardiomyocytes or HuMSC-EVs were lysed with radioimmunoprecipitation assay (RIPA) lysis buffer prior to whole-protein purification. The protein concentration was measured by a bicinchoninic acid assay (BCA) kit (Beyotime Institute of Biotechnology, Haimen, China). Equal amounts of protein underwent 10% sodium dodecyl sulfate–polyacrylamide gel electrophoresis (SDS-PAGE) and electrophoretically transferred to polyvinylidene difluoride (PVDF) membranes (EMD Millipore, Billerica, MA, USA). The membranes were incubated with the primary antibody overnight at 4°C and then incubated with a horseradish peroxidase-conjugated goat anti-rabbit IgG secondary antibody (1:2000, Ca#BE0101, EASYBIO, Beijing, China) for 1 h at room temperature. Specific protein bands were observed with an ECL Plus chemiluminescence kit (EMD Millipore) and quantitatively analyzed using Image-Pro Plus 6.0 software (Media Cybernetics, Inc., Rockville, MD, USA). Experiments were carried out in triplicate. The primary antibodies used in this study were as follows: Bax (1:5000, Ca #ab32503, Abcam, Cambridge, MA, USA), Bcl-2 (1:2000, Ca #ab196495, Abcam); cleaved-caspase 3 (1:1000, Ca#184787, Abcam), and β-actin (1:5000, Ca#0035, Abways, Shanghai, China).

### CircRNA Identification by RNA-Seq

Total RNA was isolated from the cardiomyocytes using Takara RNAiso Plus (Total RNA Extraction Reagent, Takara, Japan) in the light of the manufacturer's protocol. The Qubit 3.0 Fluorometer (Invitrogen, Carlsbad, CA, USA), and Agilent 2100 Bioanalyzer (Applied Biosystems, Carlsbad, CA, USA) were used to evaluate the concentration and integrity of the RNA, respectively. The RNA-seq library was prepared with ~1 μg of total RNA using a KAPA RNA HyperPrep Kit with RiboErase (H/M/R) for Illumina® (Kapa Biosystems, Inc., Woburn, MA, USA). After the depletion of ribosomal RNA, the ribominus RNAs were fragmented and the first strand and directional second strand were synthesized. Afterward, A-tailing and adapter ligation were performed with purified cDNA. After that, the purified, adapter-ligated DNA was amplified. The DNA library quality and concentration were evaluated with a DNA 1000 chip using an Agilent 2100 Bioanalyzer (Applied Biosystems, Carlsbad, CA, USA). Accurate quantification for sequencing applications was performed using the qPCR-based KAPA Biosystems Library Quantification kit (Kapa Biosystems, Inc., Woburn, MA). Each library was diluted to a final concentration of 10 nM and pooled in equimolar amounts before clustering. Sequencing was carried out in a 150-bp paired-end run (PE150) using the Novaseq 6000 system (Illumina, San Diego, CA, USA).

### Bioinformatics Analysis

It has been proven that circRNAs can sponge miRNAs and indirectly regulate the translation of mRNAs. To further illuminate the role of circRNAs in the protective effect of H/R injury in cardiomyocytes, the differentially expressed circRNAs (DECs) were selected to predicted the target miRNA by using miRanda software. The top 10 upregulated circRNAs with the largest fold changes (FCs) were then picked out to create a circRNA-miRNA network diagram. The functions of the parental genes consistent with the upregulated circRNAs were predicted by gene ontology (GO). GO terms with a *p* < 0.05 were chosen and integrated using Venn analysis. The top 18 enriched GO terms among the groups were ranked by fold enrichment, and the enrichment scores were presented. The pathway clusters were analyzed on the basis of the Kyoto Encyclopedia of Genes and Genomes (KEGG). According to the threshold of *P* < 0.05 and overlap gene count ≥1, significant correlations between the parental genes of upregulated circRNAs and their potential pathways were determined. The -log10 (*P*-value) indicates the significance among differentially expressed RNAs.

### Quantitative Real-Time Polymerase Chain Reaction (qRT-PCR)

Total RNA from cardiomyocytes and HuMSC-EVs were reverse-transcribed to cDNA using the Geneseed® II First Strand cDNA synthesis kit (Geneseed, Guangzhou, China). CircRNA primer sequences were designed *via* Primer Premier 5.0 and synthesized by Geneseed Biotech Co., Ltd. (Guangzhou, China). qRT-PCR was then performed with the SYBR Green qPCR Master Mix (Geneseed). The fluorescence signal emitted was detected and analyzed using the Applied Biosystems 7500 Fast Real-Time PCR System. Glyceraldehyde 3-phosphate dehydrogenase (GAPDH) was used as the control to normalize the circRNA expression data. The 2–ΔΔCt method was used to analyzed the relative expression levels. All experiments were performed in triplicate. The primer sequences of the circRNAs for qRT-PCR are listed in Table 1.

**Table 1:** The characteristics and primers of the ten circRNAs.

**Number**	**circbaseID**	**Gene**	**Sequence (5^**′**^->3^**′**^) (Forward/Reverse)**	**Product length**
1	hsa_circ_0008199	ATXN10	CATCTCCAATGTGGCCAATG	103
			CTGGGTGCTGTTTCTCTTGT	
2	hsa_circ_0007928	DCUN1D4	N/A	
3	hsa_circ_0110664	PRDM2	AAATCTAGAGAGCGGAGTGG	136
			CTGGGATTTTTTCTTCCCTG	
4	hsa_circ_0023919	PICALM	GTAGCAAGTACATGGGGAGG	105
			CTGCTTGCAGCTGTAGAATC	
5	hsa_circ_0002190	KLHDC10	TCTACCAGAAGAGAGTTGTG	131
			GAGCAAAGCCCATCTCTTAT	
6	hsa_circ_0001988	ATXN7	GGGCTCTGTCGGGAAGATC	112
			TTCTTTGGAGGGTAGGCCAA	
7	hsa_circ_0003203	SBDS	GACCATACACCGTGATCCTT	100
			CATCGAGGTCTTTTTCCCTG	
8	hsa_circ_0001971	FAM126A	CTGTGTCAAATTTGTTCAAGACA	115
			TGTGGCTCCTGGATAACTTT	
9	hsa_circ_0039400	FTO	GAGAATGGCATGCCAGATGA	143
			GCCAACTGACAGCGTTGTAA	
10	hsa_circ_0001746	MKLN1	GAAAAGGCTGTAAATGGAAC	114
			CAGGCCTTTCGAGCTTTAGA	

### Statistical Analysis

Data were analyzed in SPSS version 20.0 software (SPSS Inc., Chicago, USA). Quantitative data are presented as the mean ± standard deviation (SD). Statistical differences between groups were analyzed with one-way analysis of variance (ANOVA), followed by Tukey's multiple-comparison test. A *p*-value < 0.05 was considered statistically significant.

## Results

### Internalization of HuMSC-EVs Into AC16 Human Cardiomyocytes

**Figure 1:**
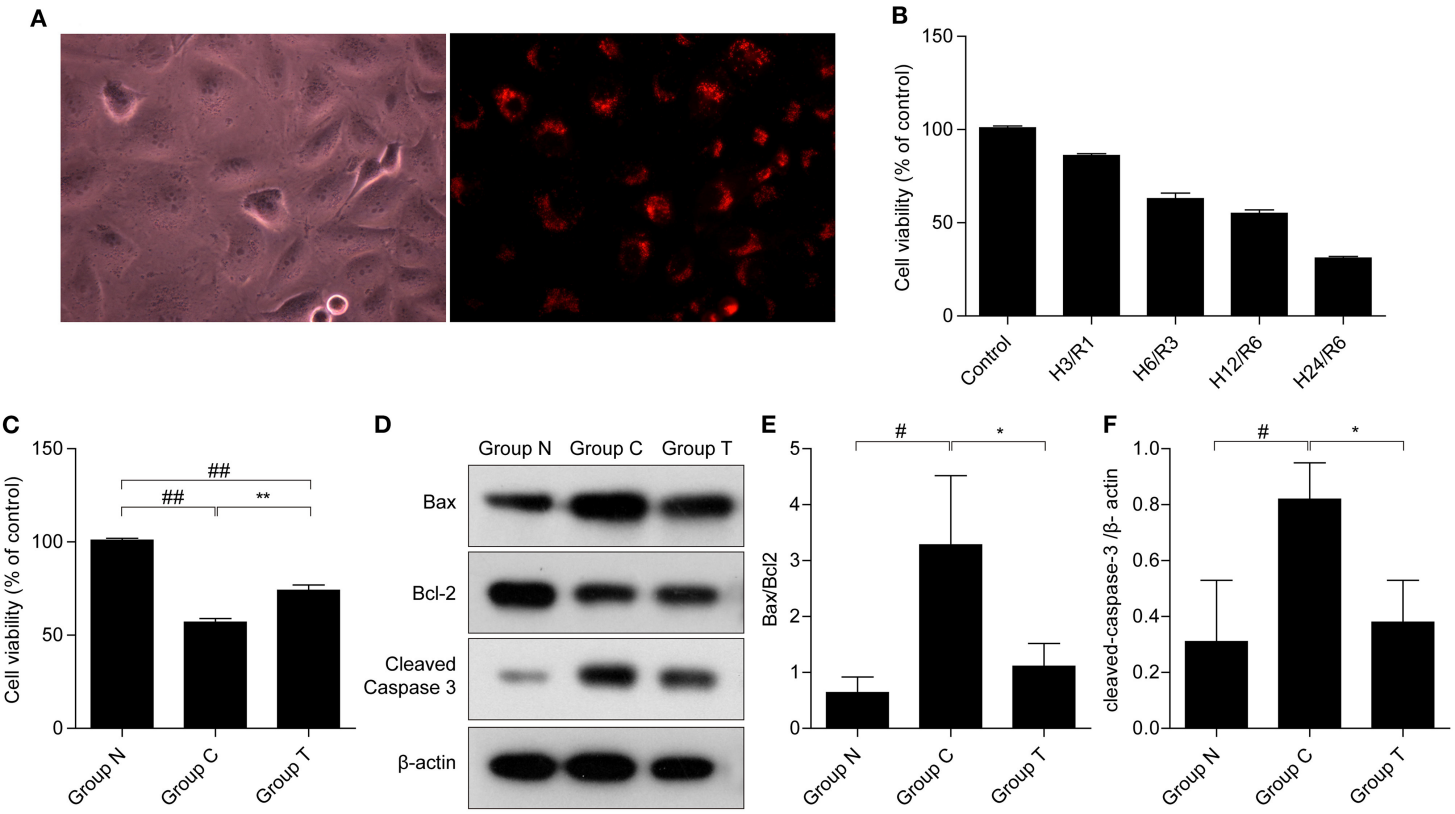
Internalization of HuMSC-EVs by AC16 human cardiomyocytes and its effects on cell survival after H/R injury. **(A)** Internalization of HuMSC-EVs by cardiomyocytes, as detected by fluorescence microscopy. **(B)** MTT was used to measure cardiomyocytes viability after exposure to H/R. **(C)** HuMSC-EVs promote survival after H/R. **(D–F)** The protein expression of cleaved-caspase 3, and Bax/bcl2 in cardiomyocytes. Values are means ± SD. ^#^*P* < 0.05 vs. Group N;^##^*P* < 0.01 vs. Group N; *P < 0.05 vs. Group C; **P < 0.01 vs. Group C. β-actin served as an internal control. H/R: hypoxia/reoxygenation. Group N: cardiomyocytes cultured under normal conditions; Group C:H/R group; Group T:H/R+HuMSC-EVs group.

To demonstrate the internalization of HuMSC-EVs by AC16 cardiomyocytes, the HuMSC-EVs were labeled with PKH-26 and incubated them with the cardiomyocytes. We observed that red fluorescence in the cytoplasm is nearly all cardiomyocytes (as shown in Figure 1A), manifesting that abundant HuMSC-EVs were internalized into the cardiomyocytes.

### HuMSC-EVs Alleviate H/R Injury

The MTT assay showed that the viability of the cardiomyocytes gradually declined in a time-dependent manner after H/R (Figure 1B), indicative of a successful H/R injury. Hypoxia for 12 h and reoxygenation for 6 h (H_12_/R_6_) resulted in a moderate injury, and this condition was selected for our subsequent experiments. As shown in Figure 1C, cell viability reduced significantly suffer to H/R injury (57% compared with group N) and HuMSC-EVs ameliorated cell viability (74% compared with group N) in cardiomyocytes subjected to H/R (^##^*p* < 0.01, vs. Group N, ^**^p < 0.01, vs. Group C). Compared to Group C, HuMSC-EV treatment upregulated Bcl-2 while downregulating the protein expression of Bax and cleaved-caspase 3 (^#^*p* < 0.05 vs. Group N; ^*^p < 0.05 vs. Group C) (as shown in Figures 1D–F). The above data suggested that HuMSC-EVs could ameliorate cell survival and mitigate apoptosis caused by H/R injury in cardiomyocytes.

### Analysis of circRNA Expression Profiles

**Figure 2:**
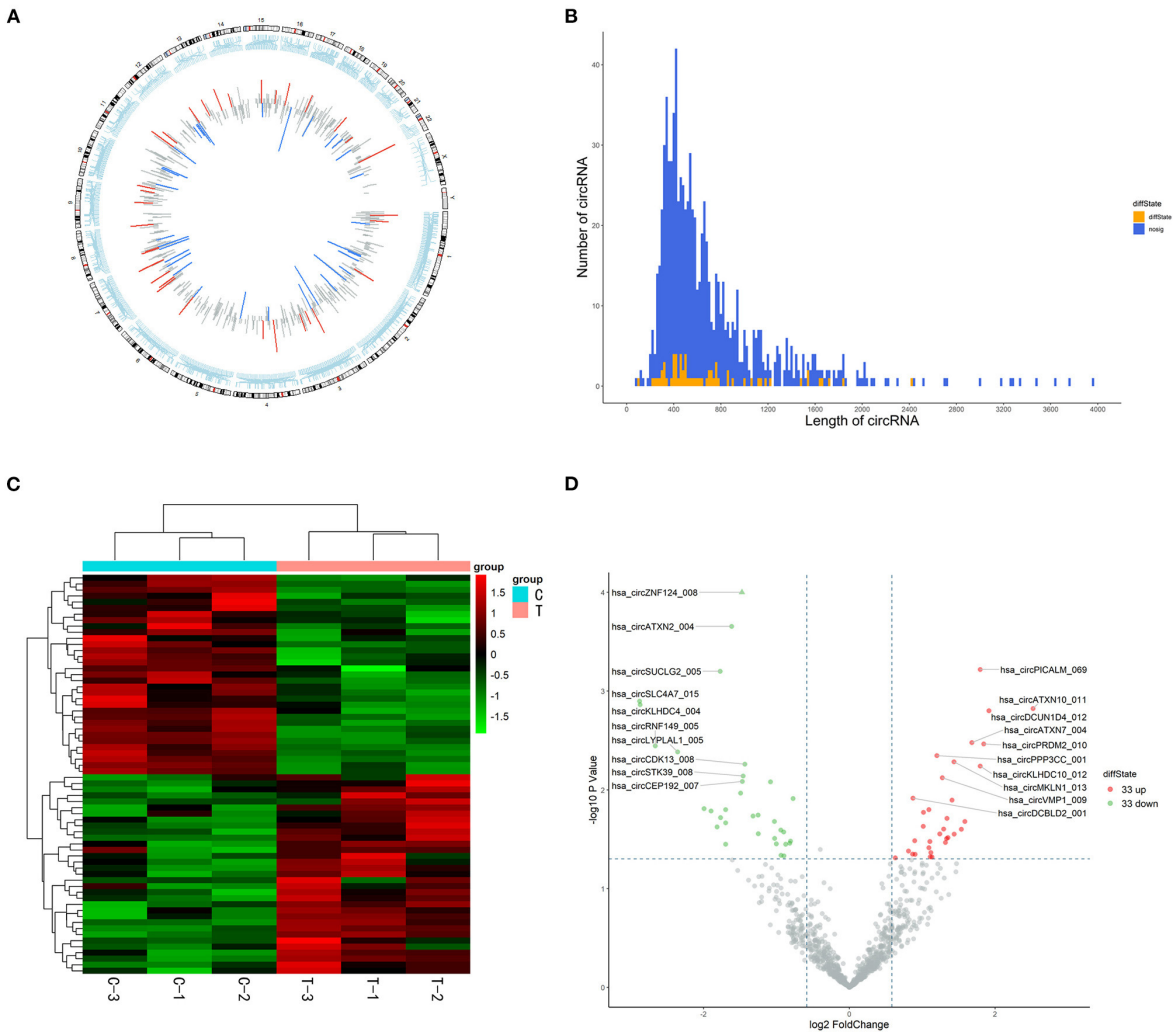
High-throughput sequencing analysis of circRNAs in cardiomyocytes**. (A)** chromosomal distribution of the of the DECs between the Group C and Group T. **(B)** Length distribution analysis of DECs, the up and downregulation circRNAs have been marked in red and green bars. **(C)** Hierarchical clustering of circRNAs. Each group contains three individuals. circRNAs are represented by single rows and samples by single columns. **(D)** Volcano plot of circRNAs. The values on the X-axes present normalized fold changes and Y-axes present P values. The color scale indicates relative expression, upregulation (red) and downregulation (green). CircRNAs with fold change > 1.5 or < −1.5, *P* < 0.05 were regarded as differentially expressed. CircRNAs: circular RNAs; DECs: differentially expressed circRNAs; Group C: H/R group; Group T: H/R+HuMSC-EVs group.

**Figure 3:**
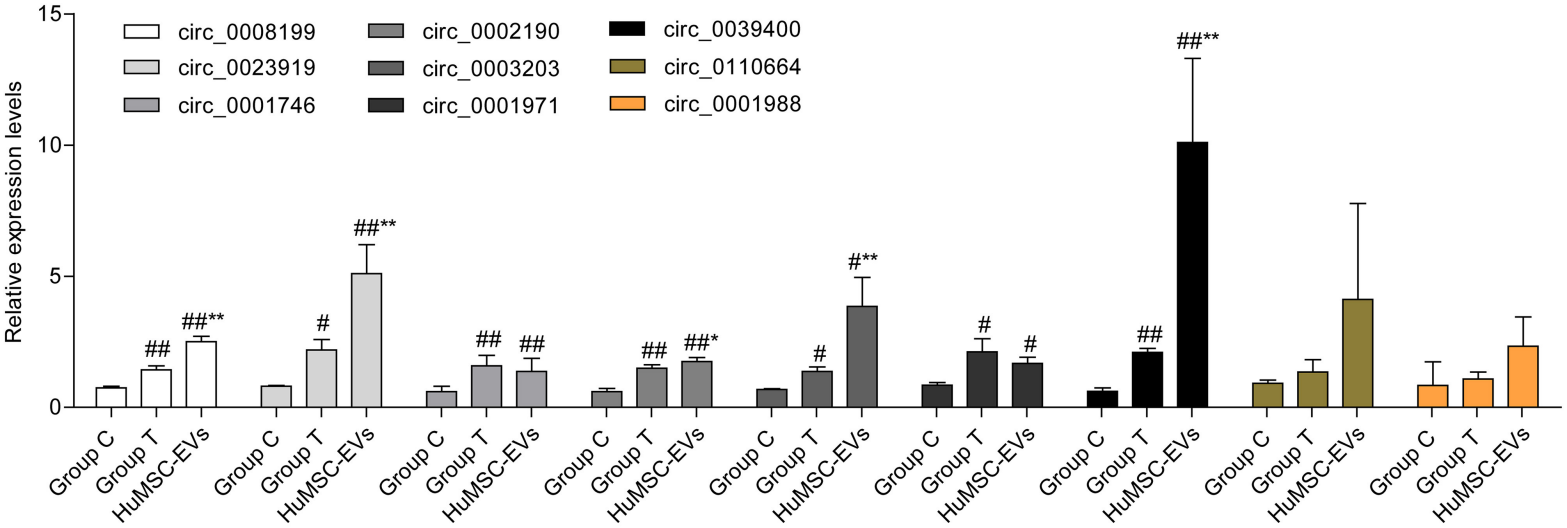
The expression of selected circRNAs (hsa_circ_0008199,hsa_circ_0110664,hsa_circ_0023919,hsa_circ_0002190,hsa_circ_0001988,hsa_circ_0003203,hsa_circ_0001971,hsa_circ_0039400,hsa_circ_0001746) in Group C, Group T, and HuMSC-EVs according to qRT-PCR. Data are shown as mean ± standard deviation (SD). #*p* < 0.05, ##*p* < 0.01 vs. Group C. *P < 0.05, **P < 0.01 vs. Group T.

In total, 15,880 circRNAs were detected in RNA-sequence analysis. About 89.49% of circRNAs were from protein-coding exons (e-circRNAs), 1.28% of circRNAs were from intronic circRNAs (i-circRNAs), and the rest were exon-intron circRNAs (e/i-circRNAs). First, we analyzed the chromosomal distribution of DECs in Groups C and T. The results indicated that these circRNAs were located at all chromosomes (Figure 2A). Sequence length analysis showed that the circRNA transcripts were mainly 400–800 bp in length (Figure 2B). A heat map and a volcano plot were used to show the DEC profiles between the two groups (Figures 2C,D). Sixty-six circRNAs were significantly differentially expressed (fold change > 1.5 or < −1.5, *p* < 0.05). Compared to Group C, 33 circRNAs were upregulated (Supplementary Table 1) and 33 circRNAs (Supplementary Table 2) were downregulated in Group T. Then, we narrowed the scope of our analysis to the 10 most aberrantly upregulated circRNAs in Group T. These 10 upregulated circRNAs, which had the largest FCs in the RNA-seq, are shown in Table 1. Furthermore, we confirmed the expression levels of the 10 circRNAs using qRT-PCR with the same samples analyzed using RNA-seq. Seven of the 10 circRNAs were confirmed to be significantly upregulated in Group T (*p* < 0.05), namely hsa_circ_0008199, hsa_circ_0023919, hsa_circ_0002190, hsa_circ_0003203, hsa_circ_0001971, hsa_circ_0039400, and hsa_circ_0001746 (Figure 3). The expression levels of these seven circRNAs were in accord with the RNA-seq analysis. Nevertheless, primers for hsa_circ_0007928 could not be designed. Additionally, the expression levels of hsa_circ_0110664 and hsa_circ_0001988 showed no differences between the two groups. The expression of the seven circRNAs was assessed in the HuMSC-EVs and was found to be similar to that in Group T (Figure 3).

**Figure 4:**
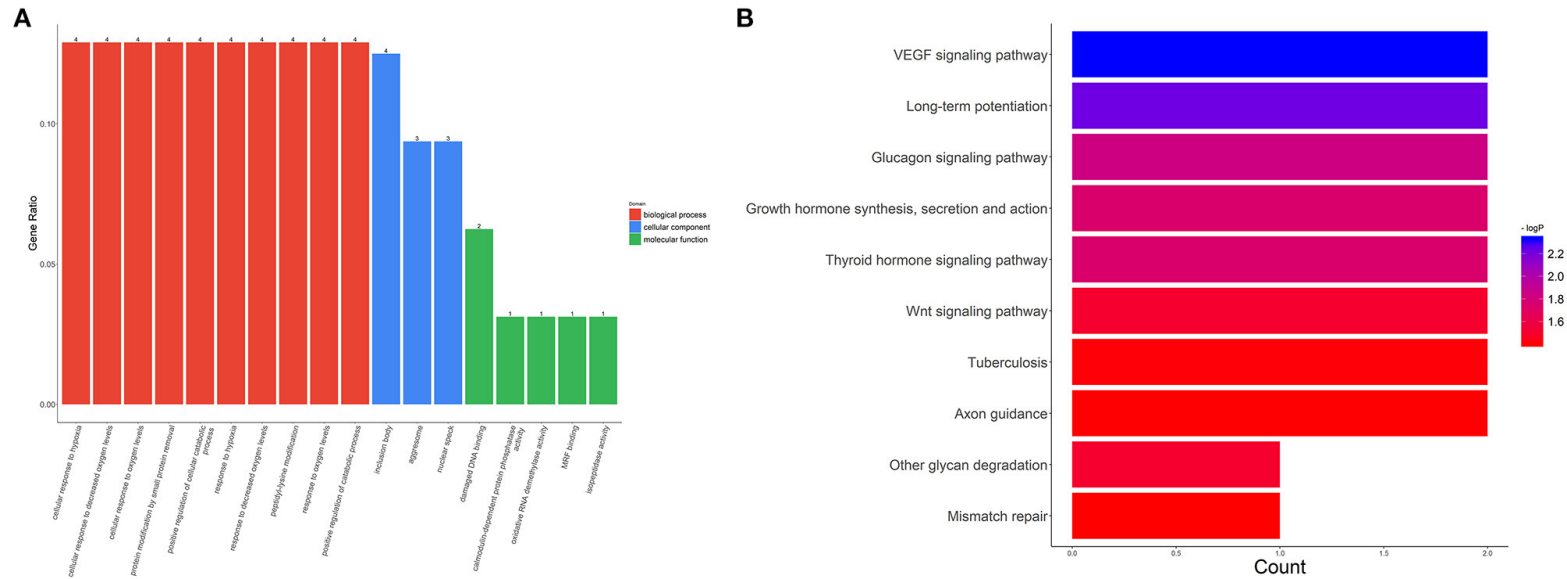
GO and KEGG analysis for circRNA-targeted mRNAs. GO enrichment for the target mRNAs **(A)** and KEGG pathway analysis **(B)**. GO: Gene Ontology; KEGG: Kyoto Encyclopedia of Genes and Genomes.

We also evaluated the functions of the upregulated circRNAs. GO analysis showed that the gene functions were mainly focused on three terms: biological processes (BP), cellular components (CC), and molecular functions (MF). Under BP, cellular response to hypoxia, cellular response to decreased oxygen levels, and cellular response to oxygen levels were the three most enriched items. CC analysis indicated that the upregulated circRNAs were significantly enriched in the inclusion body, aggresome, and nuclear speck. Under MF, damaged DNA binding, calmodulin-dependent protein phosphatase activity, and oxidative RNA demethylase activity were the three most enriched items (Figure 4A). KEGG analysis suggested that the upregulated circRNAs could target genes associated with several biological pathways, including the vascular endothelial growth factor (VEGF) signaling pathway, long-term potentiation, and the glucagon signaling pathway (Figure 4B).

### Prediction of circRNA-miRNA-mRNA

**Figure 5:**
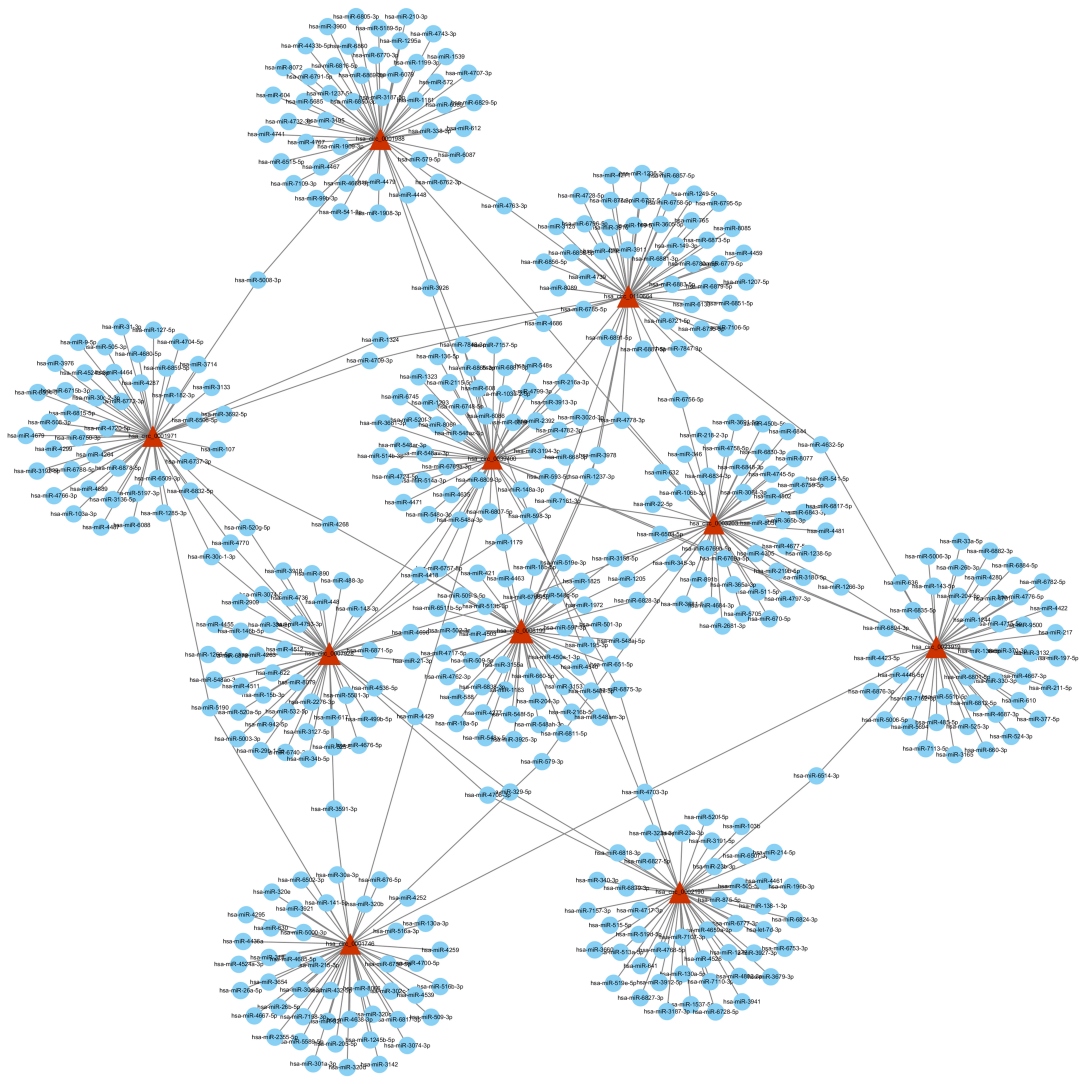
circRNA-miRNA network diagram. The triangle nodes and circular nodes represent circRNA and target miRNAs, respectively. CircRNAs: circular RNAs. miRNA: microRNA.

**Figure 6:**
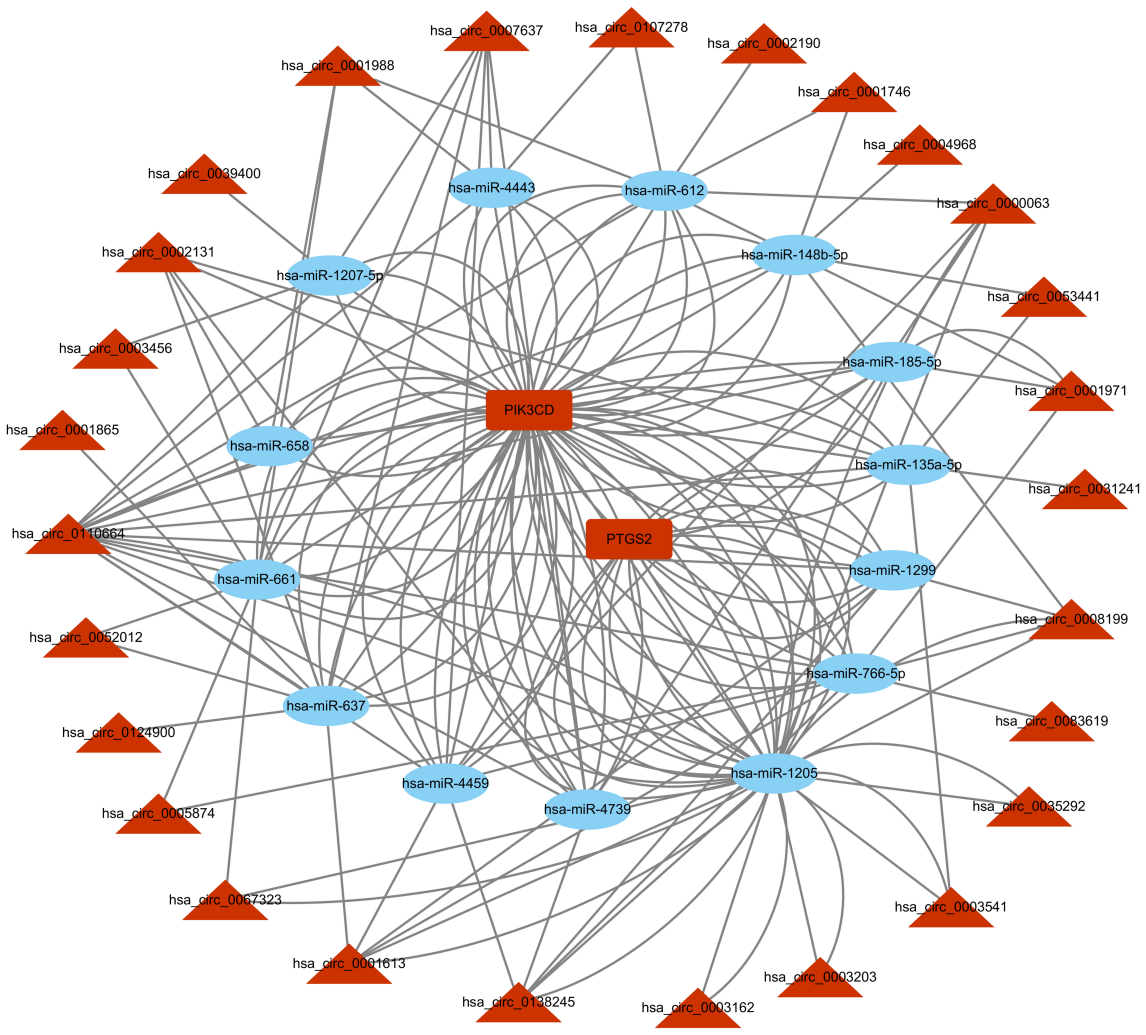
circRNA-miRNA-VEGF signal pathway network diagram. CircRNAs: circular RNAs. miRNA: microRNA.

Based on the competitive endogenous RNA (ceRNA) theory, the first 10 most aberrantly upregulated circRNAs were chosen to construct a circRNA-miRNA network diagram. As shown in Figure 5, the network diagram contained 10 circRNAs and 2070 miRNAs. To investigate whether the VEGF signaling pathway is regulated by ncRNAs, we predicted the targeted miRNAs in the VEGF signal pathway using multiMiR and identified 14 miRNAs that targeted two genes involved in the VEGF signal pathway, namely PIK3CD and PTGS2 (Figure 6).

## Discussion

Myocardial ischemia/reperfusion injury is characterized by cardiac damage as a result of restriction and subsequent restoration of blood supply to myocardium. Accumulation of reactive oxygen species (ROS) production and endoplasmic reticulum (ER) stress prominently contribute to the cell death in myocardial ischemia/reperfusion injury (20).Our previous research indicated that HuMSC-EVs could protect cardiac cells against H/R injury. MSC-derived EVs are considered to promote the repair of damaged tissues by forming abundant bioactive compounds such as microRNA and lncRNA (21). In this study, circRNAs are found abundantly expressed and upregulated during the repair of cardiomyocytes by co-cultured HuMSC-EVs following H/R injury. This result suggests that circRNAs may play a vital role in the therapeutic effect of HuMSC-EVs on cardiomyocytes after H/R injury.

Previous studies have suggested that circRNAs are related to tissue repair. Sun et al. (22) used microarray analysis to identify nine key circRNAs that were significantly increased during the Wharton's jelly-derived mesenchymal stem cell repair of damaged human endometrial stromal cells. Wu et al. (23) also reported 29 upregulated and 34 downregulated circRNAs in heart tissue following heart failure induced by myocardial infarction. In another study, Li et al. (24) used RNA-seq analysis to identify six significantly altered circRNAs during the repair of liver injury. Collectively, these data show that circRNAs act as key regulators of the biological and pathogenic process of damaged tissue repair. However, whether circRNAs play a part in the repair of cardiomyocytes that suffer H/R injury remains unclear.

High-throughput RNA sequencing was employed to screen for DECs following culture with or without HuMSC-EVs. Sixty-six significant DECs were detected, 33 of which were upregulated, and 33 of which were downregulated. Then we paid attention to the 10 upregulated circRNAs in Group T with the largest FCs in expression. Seven of these 10 circRNAs were abundantly expressed in the HuMSC-EVs. We also found that PKH26-labeled HuMSC-EVs were internalized by cardiomyocytes. Therefore, it would be logical to conclude that the circRNAs were delivered from the HuMSC-EVs to the cardiomyocytes, leading to the elevated expression of circRNAs in the cardiomyocytes.

GO and KEGG pathway analyses were carried out to investigate the biological function and relevant pathways of the upregulated circRNAs. In this study, important biological functions, such as cellular response to hypoxia, cellular response to decreased oxygen levels, and cellular response to oxygen levels determined through GO analysis, as well as significant pathways such as the VEGF signaling pathway and glucagon signaling pathway estimated from KEGG pathway analysis, were implicated in several physiological and pathophysiological activities related to anoxia. For example, VEGF is an angiogenic growth factor that can stimulate the proliferation, differentiation, and survival of vascular endothelial cells. Several experiments have indicated that VEGF plays a cardio-protective role in myocardial I/R injury (25, 26). Further, most of the host genes of the seven validated circRNAs, such as ATXN10, FAM126A, SBDS, PICALM, and FTO, are closely associated with cell survival, cell proliferation, and cell differentiation (27–31). These results indicated that the upregulated circRNAs were derived from cell development genes related to cell proliferation, which may play an important role in the cardio-protection of HuMSC-EVs.

CircRNAs may regulate the function of miRNAs by acting as ceRNAs (32). We found that the VEGF signaling pathway was involved in the cardio-protection of HuMSC-EVs. Therefore, we constructed a circRNA-miRNA-VEGF signaling pathway regulatory network for upregulated circRNAs based on the ceRNA theory (Figure 6). The results showed that two upregulated circRNA-targeted mRNAs were differentially expressed. PTGS-2, one of the circRNA-targeted mRNAs, also called cyclooxygenase (COX)-2, was increased significantly in response to a variety of stimuli, including IR injury (33).Further investigation of the gene function revealed that PIK3CD, another circRNA-targeted mRNAs, has been reported to play a protective role in renal ischemia-reperfusion injury *via* activating the PI3K/Akt signaling pathway (34). Combining our previous study results, we propose that the upregulated circRNAs may act as miRNA sponges and release PIK3CD before activating the PI3K/Akt signaling pathway. However, confirmation of the circRNA/miRNA/PIK3CD association and the function and molecular mechanism of circRNAs in the repair of H/R injury by HuMSC-EVs remain indistinct and require further research.

## Conclusions

The present study was the first to determine the expression profile of circRNAs in cardiomyocyte repair of H/R injury mediated by HuMSC-EVs. Several circRNAs may take part in biological pathways for the protection of cardiomyocytes through diverse regulatory mechanisms. Interactions of circRNA-miRNA-PIK3CD are involved in the protection of cardiomyocytes. Our present study provide a clearer understanding of the expression profile of circRNA during HuMSC-EV-mediated protection of cardiomyocytes and supply a new perspective for the treatment of H/R injury with HuMSC-EVs.

## Data Availability Statement

The datasets generated for this study can be found in online repositories. The names of the repository/repositories and accession number(s) can be found in the article/Supplementary Material.

## Ethics Statement

The studies involving human participants were reviewed and approved by Institutional Review Board of Shantou University Medical College (Shantou, China). The patients/participants provided their written informed consent to participate in this study.

## Author Contributions

CZ, JL, and LM designed the study, supervised the data collection, and analyzed the data. CZ and HW interpreted the data and prepared the manuscript for publication. All authors have read and approved the manuscript.

## Conflict of Interest

The authors declare that the research was conducted in the absence of any commercial or financial relationships that could be construed as a potential conflict of interest.

## Abbreviations

HuMSC: human umbilical cord mesenchymal stromal cell
H/R: hypoxia/reoxygenation
I/R: ischemia/reperfusion
MSCs: mesenchymal stem cells
EVs: extracellular vesicles
circRNA: Circular RNA
ceRNA: competitive endogenous RNA
FBS: fetal bovine serum
ATCC: American Type Culture Collection
OD: optical density
PVDF: polyvinylidene difluoride
DECs: differentially expressed circRNAs
GO: Gene Ontology
KEGG: Kyoto Encyclopedia of Genes and Genomes
SD: standard deviation
ANOVA: analysis of variance
VEGF: vascular endothelial growth factor.
