# Supplementary material for: Circular RNA Involvement in the Protective Effect of Human Umbilical Cord Mesenchymal Stromal Cell-Derived Extracellular Vesicles Against Hypoxia/Reoxygenation Injury in Cardiac Cells
Source: Front Cardiovasc Med. 2021 Feb 23;8:626878. doi: 10.3389/fcvm.2021.626878 (PMC7940380; doi:10.3389/fcvm.2021.626878)
Supplement: Supplementary file 1 [file Table_1.DOCX]

Table S1

| Chr_Start_End_Strand | circBankID | circbaseID | Gene | startEndRegion |
| --- | --- | --- | --- | --- |
| chr22_46085591_46136418_+ | hsa_circATXN10_011 | hsa_circ_0008199 | ATXN10 | exon-exon |
| chr4_52729602_52752804_+ | hsa_circDCUN1D4_012 | hsa_circ_0007928 | DCUN1D4 | exon-exon |
| chr1_14099572_14109326_+ | hsa_circPRDM2_010 | hsa_circ_0110664 | PRDM2 | exon-exon |
| chr11_85707868_85714494_- | hsa_circPICALM_069 | hsa_circ_0023919 | PICALM | exon-exon |
| chr7_129760588_129762042_+ | hsa_circKLHDC10_012 | hsa_circ_0002190 | KLHDC10 | exon-exon |
| chr3_63884074_63898901_+ | hsa_circATXN7_004 | hsa_circ_0001988 | ATXN7 | exon-exon |
| chr7_66458203_66459328_- | hsa_circSBDS_004 | hsa_circ_0003203 | SBDS | exon-exon |
| chr7_23015828_23023664_- | hsa_circFAM126A_008 | hsa_circ_0001971 | FAM126A | exon-exon |
| chr16_53907697_53968021_+ | hsa_circFTO_016 | hsa_circ_0039400 | FTO | exon-exon |
| chr7_131060182_131084192_+ | hsa_circMKLN1_013 | hsa_circ_0001746 | MKLN1 | exon-exon |
| chr15_52161413_52194233_+ | hsa_circTMOD3_009 | hsa_circ_0035292 | TMOD3 | exon-exon |
| chr2_32602655_32620661_+ | hsa_circBIRC6_011 | hsa_circ_0053441 | BIRC6 | exon-exon |
| chr13_95813442_95822882_- | hsa_circABCC4_037 | hsa_circ_0006659 | ABCC4 | exon-exon |
| chr8_141828375_141874498_- | hsa_circPTK2_047 | hsa_circ_0008345 | PTK2 | exon-exon |
| chr6_76344422_76388643_+ | hsa_circSENP6_018 | hsa_circ_0001613 | SENP6 | exon-exon |
| chr1_41578954_41608784_- | hsa_circSCMH1_017 | hsa_circ_0000063 | SCMH1 | exon-exon |
| chr17_57808781_57851305_+ | hsa_circVMP1_009 | hsa_circ_0107278 | VMP1 | exon-intron |
| chr19_50902107_50902741_+ | hsa_circPOLD1_003 | hsa_circ_0052012 | POLD1 | exon-exon |
| chr8_22332466_22333137_+ | hsa_circPPP3CC_001 | hsa_circ_0083619 | PPP3CC | exon-exon |
| chr3_129546645_129551669_- | hsa_circTMCC1_026 | hsa_circ_0067323 | TMCC1 | exon-exon |
| chr11_110007387_110030215_+ | hsa_circZC3H12C_007 | hsa_circ_0003541 | ZC3H12C | exon-exon |
| chr13_31204971_31205611_+ | hsa_circUSPL1_006 | hsa_circ_0005874 | USPL1 | exon-exon |
| chr4_103635594_103651893_- | hsa_circMANBA_008 | hsa_circ_0124900 | MANBA | exon-intron |
| chr9_86292641_86293514_- | hsa_circUBQLN1_018 | hsa_circ_0001865 | UBQLN1 | exon-exon |
| chr10_7409610_7423911_- | hsa_circSFMBT2_004 | hsa_circ_0017648 | SFMBT2 | exon-exon |
| chr12_116668337_116675510_- | hsa_circMED13L_009 | hsa_circ_0000443 | MED13L | intron-exon |
| chr7_33185853_33217203_+ | hsa_circBBS9_002 | hsa_circ_0003162 | BBS9 | exon-exon |
| chr8_26248758_26265892_+ | hsa_circBNIP3L_003 | hsa_circ_0002131 | BNIP3L | exon-exon |
| chr14_23375403_23380612_- | hsa_circRBM23_005 | hsa_circ_0031241 | RBM23 | exon-exon |
| chr3_98600383_98600611_- | hsa_circDCBLD2_001 | hsa_circ_0004968 | DCBLD2 | exon-exon |
| chr9_139341306_139342613_- | hsa_circSEC16A_009 | hsa_circ_0138245 | SEC16A | exon-exon |
| chr16_3900297_3901010_- | hsa_circCREBBP_007 | hsa_circ_0007637 | CREBBP | exon-exon |
| chr20_60714130_60716000_- | hsa_circPSMA7_004 | hsa_circ_0003456 | PSMA7 | exon-exon |

Table S2

| Chr_Start_End_Strand | circBankID | circbaseID | Gene | startEndRegion |
| --- | --- | --- | --- | --- |
| chr4_73956383_73958017_- | hsa_circANKRD17_046 | hsa_circ_0007883 | ANKRD17 | exon-exon |
| chr12_78443772_78452895_+ | hsa_circNAV3_046 | hsa_circ_0002419 | NAV3 | exon-exon |
| chr20_36685933_36694658_+ | hsa_circRPRD1B_004 | hsa_circ_0007365 | RPRD1B | exon-exon |
| chr15_59204761_59209198_- | hsa_circSLTM_007 | hsa_circ_0000605 | SLTM | exon-exon |
| chr2_63206322_63223901_+ | hsa_circEHBP1_019 | hsa_circ_0005552 | EHBP1 | exon-exon |
| chr12_95602618_95605043_- | hsa_circFGD6_001 | hsa_circ_0099549 | FGD6 | exon-exon |
| chr6_76412360_76412788_+ | hsa_circSENP6_051 | hsa_circ_0001614 | SENP6 | exon-exon |
| chr1_225140371_225161855_+ | hsa_circDNAH14_002 | hsa_circ_0016600 | DNAH14 | exon-exon |
| chrX_44383247_44386611_- | hsa_circFUNDC1_002 | hsa_circ_0007290 | FUNDC1 | exon-exon |
| chr1_58971731_59002413_- | hsa_circOMA1_010 | hsa_circ_0000072 | OMA1 | exon-exon |
| chr21_30698379_30702014_+ | hsa_circBACH1_012 | hsa_circ_0061395 | BACH1 | exon-exon |
| chr8_61653817_61655656_+ | hsa_circCHD7_003 | hsa_circ_0084582 | CHD7 | exon-exon |
| chr20_3888572_3893281_+ | hsa_circPANK2_004 | hsa_circ_0006873 | PANK2 | exon-exon |
| chr10_112356155_112358048_+ | hsa_circSMC3_016 | hsa_circ_0000260 | SMC3 | exon-exon |
| chr12_101316003_101336313_+ | hsa_circANO4_009 | hsa_circ_0007851 | ANO4 | exon-exon |
| chr7_40027197_40041630_+ | hsa_circCDK13_008 | hsa_circ_0001699 | CDK13 | exon-exon |
| chr2_168994597_169038600_- | hsa_circSTK39_008 | hsa_circ_0002029 | STK39 | exon-exon |
| chr18_12999419_13030607_+ | hsa_circCEP192_007 | hsa_circ_0107922 | CEP192 | exon-exon |
| chr1_247319707_247323115_- | hsa_circZNF124_008 | hsa_circ_0112879 | ZNF124 | exon-exon |
| chr10_31644072_31676727_+ | hsa_circZEB1_007 | hsa_circ_0007045 | ZEB1 | exon-exon |
| chr12_111991961_111993723_- | hsa_circATXN2_004 | hsa_circ_0000439 | ATXN2 | exon-exon |
| chr10_120832401_120833449_- | hsa_circEIF3A_003 | hsa_circ_0004350 | EIF3A | exon-exon |
| chr5_14293120_14336836_+ | hsa_circTRIO_037 | hsa_circ_0007638 | TRIO | exon-exon |
| chr7_91924202_91981956_+ | hsa_circANKIB1_009 | hsa_circ_0002744 | ANKIB1 | exon-exon |
| chr7_116339124_116340338_+ | hsa_circMET_002 | hsa_circ_0082002 | MET | exon-exon |
| chr3_67568670_67579610_- | hsa_circSUCLG2_005 | hsa_circ_0006429 | SUCLG2 | exon-exon |
| chr11_85718584_85742653_- | hsa_circPICALM_018 | hsa_circ_0023936 | PICALM | exon-exon |
| chr8_15508205_15531345_+ | hsa_circTUSC3_007 | hsa_circ_0006410 | TUSC3 | exon-exon |
| chr8_28866588_28876430_+ | hsa_circHMBOX1_013 | hsa_circ_0083826 | HMBOX1 | exon-exon |
| chr1_219366423_219392054_+ | hsa_circLYPLAL1_005 | hsa_circ_0004314 | LYPLAL1 | exon-intergenic |
| chr2_101898320_101911643_- | hsa_circRNF149_005 | hsa_circ_0055904 | RNF149 | exon-exon |
| chr16_87782278_87795646_- | hsa_circKLHDC4_004 | hsa_circ_0000724 | KLHDC4 | exon-exon |
| chr3_27478878_27490288_- | hsa_circSLC4A7_015 | hsa_circ_0006215 | SLC4A7 | exon-exon |
